# Invisible Until It Burst: Unexpected Subarachnoid Hemorrhage From a Rapid-Onset Infectious Aneurysm in a Patient With Endocarditis

**DOI:** 10.7759/cureus.81843

**Published:** 2025-04-07

**Authors:** Tatsuya Tanaka, Talgat Tilyeubyek, Furitsu Shimada, Yuki Takeuchi, Akira Matsuno

**Affiliations:** 1 Department of Neurosurgery, International University of Health and Welfare Narita Hospital, Narita, JPN; 2 Department of Gastroenterology, Kouhoukai Takagi Hospital, Okawa, JPN

**Keywords:** brain mri, cerebral infarction, colon cancer, infectious intracranial aneurysm, infective endocarditis, rapid aneurysm formation, streptococcus sanguinis, subarachnoid hemorrhage, vegetation

## Abstract

Infective endocarditis (IE) can lead to serious neurological complications, including septic embolism and infectious intracranial aneurysms (IIAs). Although IIAs are rare, their rupture often results in catastrophic outcomes. Predicting their formation, especially within a short period, remains a clinical challenge.

We present the case of a man in his 70s who was newly diagnosed with colon cancer. During preoperative evaluation, transthoracic echocardiography revealed vegetations on the aortic and mitral valves, leading to a diagnosis of IE caused by Streptococcus sanguinis. On the third day of hospitalization, the initial brain magnetic resonance imaging (MRI) revealed asymptomatic cerebral infarction, but magnetic resonance angiography (MRA) did not show any aneurysms. Despite appropriate antibiotic therapy, the patient developed sudden left hemiparesis and impaired consciousness on day 6. Emergent computed tomography (CT) and computed tomography angiography (CTA) revealed a subarachnoid hemorrhage and a newly formed ruptured aneurysm in the M1 segment of the middle cerebral artery. Given the patient's overall prognosis, neurosurgical intervention was deemed inappropriate, and best supportive care was initiated. The patient passed away shortly thereafter.

This case highlights the unpredictable nature of IIAs in IE. Although imaging performed just three days prior showed no aneurysms, a rapidly formed and ruptured IIA resulted in fatal subarachnoid hemorrhage. It underscores the challenge of predicting the rupture of infectious aneurysms in IE and emphasizes the importance of frequent imaging follow-up, even when initial imaging findings are normal.

## Introduction

Infective endocarditis (IE) remains a life-threatening disease characterized by microbial infection of the endocardial surface of the heart, frequently leading to systemic complications due to septic embolization [1,2]. Among these, neurological events occur in up to 30%-40% of cases, with cerebral infarction being the most common [1,2]. Although rare, infectious intracranial aneurysms (IIAs) are among the most feared complications due to their high risk of rupture and resulting morbidity and mortality [3-5].

IIAs typically arise when septic emboli lodge in cerebral arteries, damaging the vascular endothelium and leading to localized infection, inflammation, and subsequent aneurysm formation [6]. Diagnosis is often delayed, as IIAs can be asymptomatic until rupture [3-5]. Furthermore, the natural history of IIA progression remains poorly defined, and there are no established guidelines for optimal surveillance or the timing of intervention [1,2,7].

Recent reports suggest that IIAs may form and rupture within a matter of days, even in patients with previously normal neurovascular imaging [3-5]. This case highlights such a scenario: a patient with IE developed fatal subarachnoid hemorrhage due to a newly formed ruptured IIA just three days after negative magnetic resonance angiography (MRA), emphasizing the diagnostic difficulties and the importance of close follow-up in high-risk patients.

## Case presentation

The patient is a male in his 70s with a history of hypertension, controlled with medication, smoking (20 cigarettes/day for 20 years), and excessive alcohol consumption (50 g/day). He presented to a local medical institution approximately two weeks prior with complaints of loss of appetite and epigastric pain. Blood tests revealed anemia, and black stools were noted. Upper and lower gastrointestinal endoscopy suggested sigmoid colon and rectal cancer, and a biopsy confirmed the diagnosis of advanced colorectal cancer. The patient was subsequently referred to our hospital for further management.

On day 1, clinical laboratory results showed an elevated white blood cell count (WBC 8,220/μL), anemia (hemoglobin 7.2 g/dL), liver dysfunction (alanine aminotransferase [ALT] 139 U/L, aspartate aminotransferase [AST] 116 U/L, and alkaline phosphatase 176 U/L), and elevated inflammatory markers (C-reactive protein [CRP] 5.75 mg/dL). The initial clinical tests are summarized in Table 1.

**Table 1:** Labs on initial presentation. MCV, mean corpuscular volume; MCHC, mean corpuscular hemoglobin concentration; RDW, red cell distribution width; MPV, mean platelet volume; BUN, blood urea nitrogen; ALT, alanine aminotransferase; AST, aspartate aminotransferase

Laboratory test	Result	Normal range
White blood cells	8,220/μL	4,000-8,000 /μL
Red blood cells	2.65 x 10^6^/μL	4.35-5.55 x 10^6^/μL
Hemoglobin	7.2 g/dL	13.7-16.8 g/dL
Hematocrit	23.70%	40.7%-550.1%
MCV	89.4 fL	83.6-98.2 fL
MCHC	30.40%	31.7%-35.3 %
RDW	15.5 fL	11.8-14.5 fL
MPV	10.7 fL	8-12 fL
Platelet count	158,000/μL	140,000-340,000/μL
Neutrophils	87%	38.5%-80.5%
Lymphocytes	6.90%	16.5%-49.5%
Monocytes	6.40%	2.0%-10.0%
Eosinophils	0.00%	0.0-8.5%
Basophils	0.10%	0.0-2.5%
Glucose	139 mg/dL	73-109 mg/dL
BUN	22 mg/dL	8.0-20.0 mg/dL
Creatinine	0.87 mg/dL	0.65-1.07 mg/dL
Sodium	139 mEq/L	138-145 mEq/L
Potassium	3.8 mEq/L	3.6-4.8 mEq/L
Chloride	108 mEq/L	101-108 mEq/L
Calcium	8.1 mg/dL	8.8-10.1 mg/dL
Protein (Total)	6.9 g/dL	6.6-8.1 g/dL
Albumin	2.9 g/dL	3.4-5.4 g/dL
Bilirubin (Total)	0.9 mg/dL	0.4-1.5 mg/dL
ALT	139 U/L	10-42 U/L
AST	116 U/L	13-30 U/L
Alkaline phosphatase	176 U/L	38-113 U/L
C-reactive protein	5.75 mg/dL	0.00-0.14 mg/dL

**Figure 1:**
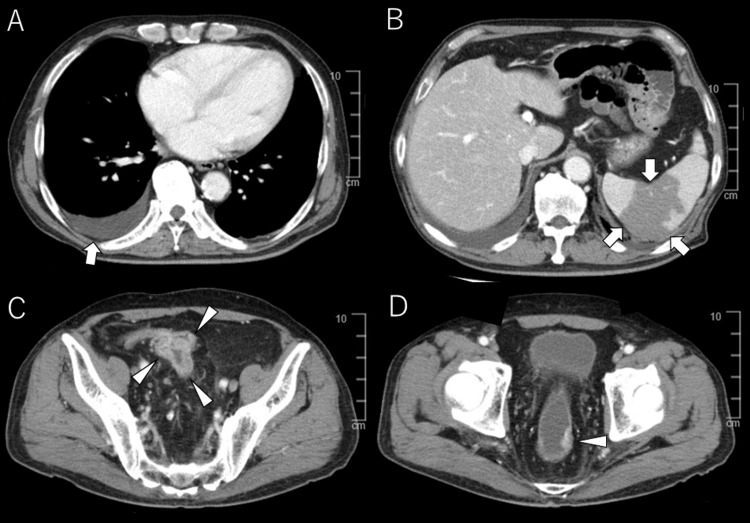
Abdominal contrast-enhanced CT. (A) Cardiomegaly with right-sided pleural effusion is observed (arrow). (B) An irregular hypodense area in the spleen is noted, suggestive of splenic infarction (arrow). (C) A localized wall thickening is observed in the sigmoid colon, with heterogeneous post-contrast enhancement, suggestive of sigmoid colon cancer (arrowhead). (D) A small protruding lesion is noted on the left wall of the rectum, consistent with rectal cancer (arrowhead).

Contrast-enhanced chest and abdominal computed tomography (CT) revealed cardiomegaly and right-sided pleural effusion (Figure 1A).

Localized wall thickening with heterogeneous enhancement in the sigmoid colon, consistent with sigmoid colon cancer, was observed (Figure 1B). Additionally, a protruding lesion on the left wall of the rectum was consistent with rectal cancer (Figure 1C). An irregular hypodense area in the spleen, suggestive of splenic infarction, was observed (Figure 1D).

**Figure 2:**
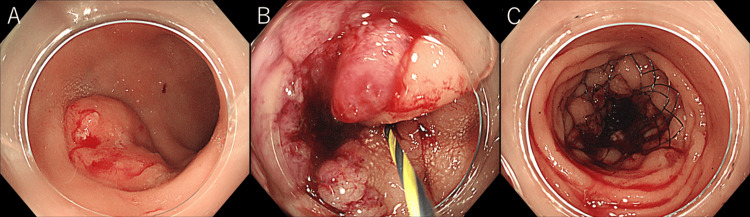
Lower gastrointestinal endoscopy. (A) A 20 mm protruding lesion is seen in the rectum. (B) Circumferential colon cancer is observed in the sigmoid colon, with narrowing of the lumen. (C) A self-expanding metal stent has been placed.

On Day 2, a self-expanding metallic stent was placed in the sigmoid colon due to circumferential narrowing caused by colon cancer, resulting in successful lumen expansion (Figure 2).

**Figure 3:**
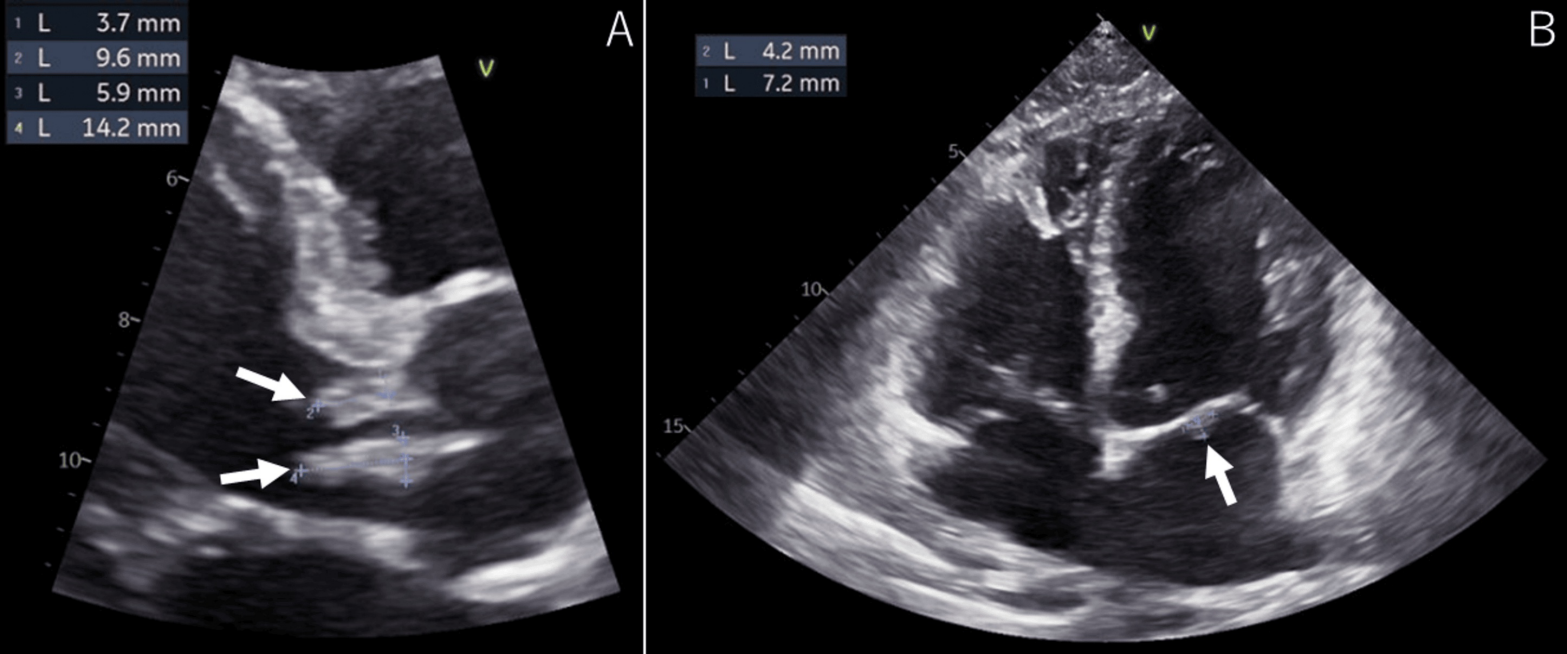
Echocardiography. (A) A club-shaped mass is observed on the aortic and mitral valves (arrow). (B) A mass is observed in the aortic valve annulus (arrow).

On Day 3, echocardiography revealed multiple club-shaped masses on the aortic and mitral valves, some exceeding 10 mm in size. The patient was diagnosed with IE complicated by severe aortic and mitral valve regurgitation (Figure 3).

**Figure 4:**
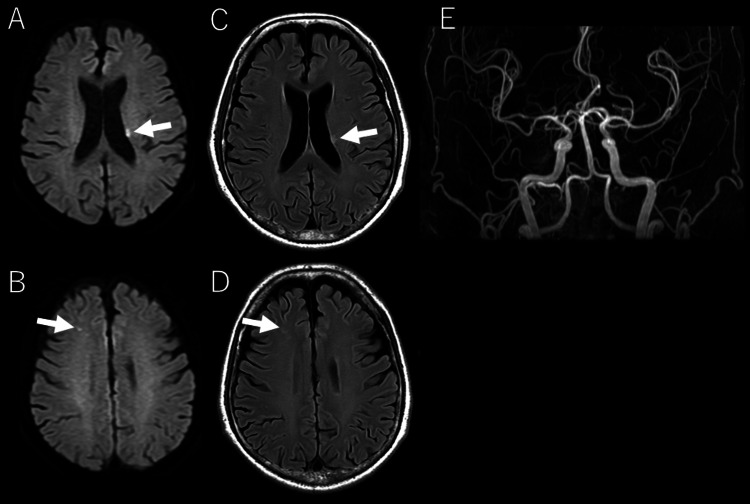
Head MRI and MRA. DWI (A, B) and FLAIR (C, D) show high signal areas in the left corona radiata and frontal lobe, consistent with acute to subacute ischemic infarction. MRA (E) reveals no significant aneurysms. DWI, diffusion-weighted imaging; FLAIR, fluid-attenuated inversion recovery; MRI, magnetic resonance imaging; MRA, magnetic resonance angiography

Empirical antimicrobial therapy with Sulbactam/Ampicillin (3 g three times daily) and Ceftriaxone (2 g once daily) was initiated. Blood cultures subsequently grew Streptococcus sanguinis. On the same day, head magnetic resonance imaging (MRI) and MRA were performed, revealing asymptomatic subacute ischemic infarction, with no aneurysms identified (Figure 4).

In the future, open-heart surgery for IE and surgery for colorectal cancer were planned. The average blood pressure from Day 1 to Day 6 was 133/67 mmHg (range: 122-140/52-78).

**Figure 5:**
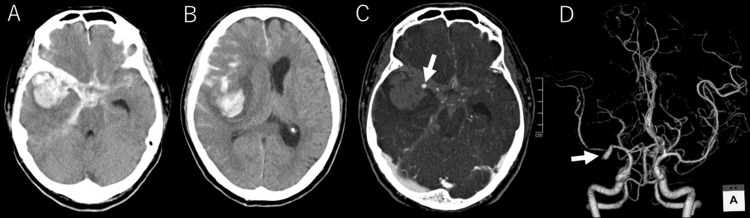
Head CT and CTA. (A, B) Subarachnoid hemorrhage is observed from the basal cistern to the suprasellar cistern, along the bilateral Sylvian fissures, and in the right hemisphere sulci. A subcortical hemorrhage is noted in the right temporal lobe. There is significant compression of the right lateral ventricle with a midline shift to the left. (C, D) A 12 x 3 mm contrast-enhancing mass is observed protruding dorsally from the proximal M1 segment of the right middle cerebral artery, consistent with a pseudoaneurysm (arrow). CTA, computed tomography angiography; CT, computed tomography

On Day 6, the patient developed altered consciousness and was found in the bathroom. Physical examination revealed left-sided hemiparesis, and the Japanese Coma Scale score was 100. Pupillary examination revealed a right pupil of 4 mm and a left pupil of 3 mm, with sluggish light reflexes. Vital signs showed a heart rate of 90 bpm, blood pressure of 130/55 mmHg, and oxygen saturation of 93% on room air. A head CT scan was promptly performed, revealing subcortical hemorrhage in the right temporal lobe, along with subarachnoid hemorrhage (SAH) associated with a thick hematoma in the right Sylvian fissure. Compression and narrowing of the right lateral ventricle with a midline shift were also noted (Figures 5A, 5B).

Three-dimensional computed tomography angiography (3D CTA) identified a newly formed 12 × 3 mm aneurysm at the proximal M1 segment of the right middle cerebral artery (Figures 5C, 5D).

Given that no aneurysm was seen on the MRA performed on Day 3, it was concluded that an IIA had formed and ruptured within a short period.

Following consultation with the neurosurgery team, several life-saving measures, including external decompression, parent vessel occlusion, and bypass surgery, were considered. However, given the progression of the colorectal cancer and IE, both life expectancy and functional prognosis were deemed extremely poor. After discussions with the patient's family, the decision was made to forgo aggressive treatment, and best supportive care was initiated. The patient's condition gradually worsened, and he passed away on Day 6.

## Discussion

IIAs are rare but severe complications of IE, with an incidence reported to range from 2% to 10% of all IE cases [7-10]. The pathophysiology of IIAs involves direct bacterial invasion of the cerebral arterial wall via bacteremia or damage caused by septic emboli, leading to vascular weakening and the subsequent formation of aneurysms [3-6]. The mortality rate for patients with unruptured IIAs is reported to range from 10% to 30%, but it can rise to as high as 80% in cases where rupture occurs [11]. Therefore, timely detection of unruptured IIAs and appropriate intervention are crucial [12].

In the present case, after the diagnosis of IE, initial imaging confirmed the absence of an aneurysm. However, within a few days, an aneurysm developed and subsequently ruptured. Previous reports have described similar cases where aneurysms ruptured unexpectedly despite initial imaging showing no aneurysm [3-5]. In these instances, patients presented with ischemic stroke, and initial imaging studies such as MRA or CTA confirmed the absence of aneurysms. However, rupture of the infectious aneurysm occurred within 38 hours to three days after the imaging. Additionally, the presence of an infectious aneurysm in a previously occluded vessel suggests that the aneurysm likely formed as a result of bacterial embolism, where bacteria invade the cerebral artery wall, causing vascular weakening and aneurysm formation [3,4,5,12]. However, while IE is often associated with ischemic stroke, only 20%-30% of these strokes are symptomatic [1,2]. The occurrence of ischemic stroke does not necessarily predict the formation of an infectious aneurysm. Although many guidelines recommend neuroimaging upon the diagnosis of IE [1,2], they also note that "vascular imaging should not be routinely performed, and CTA or MRA may be sufficient for screening in cases where an infectious aneurysm is suspected. For patients diagnosed with an infectious aneurysm on CTA or MRA, those with acute cerebral hemorrhage, those in whom non-invasive techniques are negative but suspicion remains, and those for whom mechanical thrombectomy is considered, catheter angiography should be performed" [1].

The findings from this case and others suggest that a single negative imaging study may not be sufficient, particularly in high-risk patients. While routine follow-up imaging for all IE patients may not be practical, a risk-based approach could prove beneficial in preventing unexpected aneurysm rupture. The risk factors for the formation and rupture of infectious aneurysms in patients with IE remain unclear, and further research is needed to better understand these mechanisms.

A limitation of this report is that a pathological examination of the aneurysm was not performed. Although arterial dissection could also have contributed to aneurysm rupture, given the patient's IE and the absence of findings suggestive of dissection on MRA and CTA, the rupture is most likely due to an infectious aneurysm.

## Conclusions

This case illustrates the rare but devastating complication of rapidly forming and rupturing IIAs in the setting of IE. Despite appropriate antimicrobial therapy and negative neurovascular imaging early in the disease course, a fatal SAH occurred within days due to the abrupt development of a ruptured aneurysm. Even when initial neuroimaging is negative, the risk of IIA should not be excluded. Clinicians should remain vigilant for the possibility of sudden neurological deterioration, even in the absence of radiographic evidence of an aneurysm, as timely detection and intervention are crucial for improving outcomes.

## References

[1] Delgado V, Ajmone Marsan N, de Waha S (2023). 2023 ESC Guidelines for the management of endocarditis. Eur Heart J.

[2] Holland TL, Baddour LM, Bayer AS, Hoen B, Miro JM, Fowler VG Jr (2016). Infective endocarditis. Nat Rev Dis Primers.

[3] Wang JL, Hinduja AP, Powers CJ (2017). Successful coil embolization of a ruptured mycotic aneurysm that developed three days after septic embolic infarction: case report and review of the literature. J Clin Neurosci.

[4] Nukata R, Ikeda H, Akaike N (2022). Short-term aneurysm formation and rupture due to septic embolism diagnosed with a thrombus retrieved from another occluded artery. Surg Neurol Int.

[5] Yanagawa T, Ikeda S, Yoshitomi S, Shibata A, Ikeda T (2023). A case of infectious intracranial aneurysm that formed and ruptured within a few days after occlusion of the proximal middle cerebral artery by infective endocarditis. Surg Neurol Int.

[6] Ducruet AF, Hickman ZL, Zacharia BE, Narula R, Grobelny BT, Gorski J, Connolly ES Jr (2010). Intracranial infectious aneurysms: a comprehensive review. Neurosurg Rev.

[7] Alawieh AM, Dimisko L, Newman S (2023). Management and long-term outcomes of patients with infectious intracranial aneurysms. Neurosurgery.

[8] Singla A, Fargen K, Blackburn S (2016). National treatment practices in the management of infectious intracranial aneurysms and infective endocarditis. J Neurointerv Surg.

[9] Duval X, Iung B, Klein I (2010). Effect of early cerebral magnetic resonance imaging on clinical decisions in infective endocarditis: a prospective study. Ann Intern Med.

[10] Calderón-Parra J, Domínguez F, González-Rico C (2024). Epidemiology and risk factors of mycotic aneurysm in patients with infective endocarditis and the impact of its rupture in outcomes. Analysis of a National Prospective Cohort. Open Forum Infect Dis.

[11] Alawieh A, Chaudry MI, Turner RD, Turk AS, Spiotta AM (2018). Infectious intracranial aneurysms: a systematic review of epidemiology, management, and outcomes. J Neurointerv Surg.

[12] Tran Hue A, Tanaka T, Yamane F, Itokawa H, Nakahara K, Matsuno A (2024). Management of unruptured infectious intracranial aneurysms in infective endocarditis: a case report and literature review. Cureus.

